# Mechanical and Biocompatibility Properties of Calcium Phosphate Bioceramics Derived from Salmon Fish Bone Wastes

**DOI:** 10.3390/ijms21218082

**Published:** 2020-10-29

**Authors:** Merve Bas, Sibel Daglilar, Nilgun Kuskonmaz, Cevriye Kalkandelen, Gokce Erdemir, Serap E. Kuruca, Dilshat Tulyaganov, Tomohiko Yoshioka, Oguzhan Gunduz, Denisa Ficai, Anton Ficai

**Affiliations:** 1 Department of Metallurgical and Materials Engineering, Faculty of Chemical and Metallurgical Engineering, Yildiz Technical University, Istanbul 34220, Turkey; meerveebaas@gmail.com (M.B.); dagli@yildiz.edu.tr (S.D.); kkonmaz@yildiz.edu.tr (N.K.); 2 Center for Nanotechnology & Biomaterials Application and Research (NBUAM), Marmara University, Istanbul 34722, Turkey; 3 Vocational School of Technical Sciences, Istanbul University-Cerrahpaşa, Istanbul 34500, Turkey; 4 Department of Molecular Medicine, Aziz Sancar Institute of Experimental Medicine, Istanbul University, Istanbul 34093, Turkey; gokcerdemir@gmail.com; 5 Department of Physiology, Istanbul Faculty of Medicine, Istanbul University, Capa Campus, Istanbul 34093, Turkey; sekuruca@istanbul.edu.tr; 6 Turin Polytechnic University in Tashkent, 17 Small Ring Street, Tashkent 100095, Uzbekistan; tulyaganovdilshat@gmail.com; 7 Graduate School of Interdisciplinary Science and Engineering in Health Systems, Okayama University, 3-1-1, Tsushima, Kita-ku, Okayama 700-8530, Japan; tomohiko.yoshioka@cc.okayama-u.ac.jp; 8 Department of Metallurgical and Materials Engineering, Faculty of Technology, Marmara University, Istanbul 34722, Turkey; 9 Department of Inorganic Chemistry, Physical Chemistry and Electrochemistry, Faculty of Applied Chemistry and Materials Science, University POLITEHNICA of Bucharest, 1-7 Gh Polizu Street, 060042 Bucharest, Romania; denisa.ficai@upb.ro; 10 National Centre for Micro- and Nanomaterials; University POLITEHNICA of Bucharest, Splaiul Independentei 313, 060042 Bucharest, Romania; 11 Department of Science and Engineering of Oxide Materials and Nanomaterials, Faculty of Applied Chemistry and Materials Science, University POLITEHNICA of Bucharest, 1-7 Gh Polizu Street, 060042 Bucharest, Romania; 12 Academy of Romanian Scientists, Ilfov st. 3, 060042 Bucharest, Romania

**Keywords:** salmon fish bone, hydroxyapatite, mechanical properties, sintering

## Abstract

Natural calcium phosphates derived from fish wastes are a promising material for biomedical application. However, their sintered ceramics are not fully characterized in terms of mechanical and biological properties. In this study, natural calcium phosphate was synthesized through a thermal calcination process from salmon fish bone wastes. The salmon-derived calcium phosphates (sCaP) were sintered at different temperatures to obtain natural calcium phosphate bioceramics and then were investigated in terms of their microstructure, mechanical properties and biocompatibility. In particular, this work is concerned with the effects of grain size on the relative density and microhardness of the sCaP bioceramics. Ca/P ratio of the sintered sCaP ranged from 1.73 to 1.52 when the sintering temperature was raised from 1000 to 1300 °C. The crystal phase of all the sCaP bioceramics obtained was biphasic and composed of hydroxyapatite (HA) and tricalcium phosphate (TCP). The density and microhardness of the sCaP bioceramics increased in the temperature interval 1000–1100 °C, while at temperatures higher than 1100 °C, these properties were not significantly altered. The highest compressive strength of 116 MPa was recorded for the samples sintered at 1100 °C. In vitro biocompatibility was also examined in the behavior of osteosarcoma (Saos-2) cells, indicating that the sCaP bioceramics had no cytotoxicity effect. Salmon-derived biphasic calcium phosphates (BCP) have the potential to contribute to the development of bone substituted materials.

## 1. Introduction

Hydroxyapatite (HA) [HA, Ca_5_(PO_4_)_3_OH] comprises ~70% by weight of bones, the remainder being organic molecules such as collagen and water [1]. Biological parts can be removed from the bone by a high-temperature calcination process, after which only HA remains as a mineral component in the structure [2]. HA can be synthetically produced from chemicals or synthesized from natural sources by different routes, including hydrothermal conversion and high-temperature calcination [3]. Calcium phosphate ceramics with HA as the principle phase is used as a filler to substitute damaged bone or as a coating on implants to support bone growth due to its similarity to the mineral structure of bones and teeth in mammals [4]. When the contribution of trace elements such as magnesium, zinc and strontium to the mechanical and biocompatible properties of bone is considered, natural HA (nHA) provides advantages because it contains the aforementioned elements in the composition. Another merit of natural resources is that access to raw materials is easier and much more economical.

A massive amount of fish and shellfish (more than 91 million tons) are hunted annually worldwide. About 50–60% of hunting is used for human consumption, while the rest is wasted [5]. For example, in salmon filleting in a typical automatic fillet line, fillets comprise about 59–63% of body weight. The remaining spine (9–15%) and head (10–12%) are byproducts [6]. There is no commercially viable application for the use of these residues, and they are accepted as a worthless byproduct. These wastes (scales, skin and bones) represent a risk to the ecosystem as they present environmental and health risks. To eliminate these potential risks, studies have been conducted to produce nHA from fish bones for commercial and biomedical applications. Converting fish bone wastes into HA is environmentally friendly and is an excellent chance to reduce costs for biomedical applications [7]. Granito et al. have reviewed fish HA for bone tissue engineering. HA from fish showed excellent biocompatibility as a result of non-cytotoxicity and superior handling properties [7]. Specifically, Shi et al. have prepared three types of nHA from rainbow trout (*Oncorhynchus mykiss*), cod (*Gadus*) and salmon (*Oncorhynchus Keta*) fish bones using thermal calcination management and examined their characterization and biocompatibility [8]. They concluded that nHA derived from rainbow trout and salmon bone materials contained essential mineral ions that improved proliferation, differentiation, cell adhesion and mineralized tissue formation [8]. Although natural calcium phosphates obtained from fish wastes are thus a promising material for biomedical applications, they need to be sintered and transformed into bulk bodies, especially for use as bone-substituted materials. However, such investigations have not sufficiently been carried out to date. In particular, the mechanical properties after sintering are an important aspect of bone replacements. In fact, Deb et al. have investigated the mechanical properties of composites of HA derived from *Puntius conchonius* fish scales and polymethylmethacrylate (PMMA) [9]. However, this study did not reveal the properties of bulk materials prepared only from natural calcium phosphates. Furthermore, the sintering process may affect the biological properties of the bulk materials.

The natural calcium phosphates were obtained from salmon fish bones by a calcination method. Then, sintered ceramic bodies were obtained from the natural calcium phosphate powders, and the effects of different sintering temperatures on the microstructure and mechanical properties of the sintered materials were investigated. In addition, biocompatibility studies were conducted to evaluate the potential of the sintered bodies as a bioactive material. These results suggest that the natural calcium phosphates derived from salmon fish bone are a promising biomaterial in the field of bone repair and regeneration applications.

## 2. Materials and Methods

### 2.1. Preparation of sCaP

**Figure 1:**
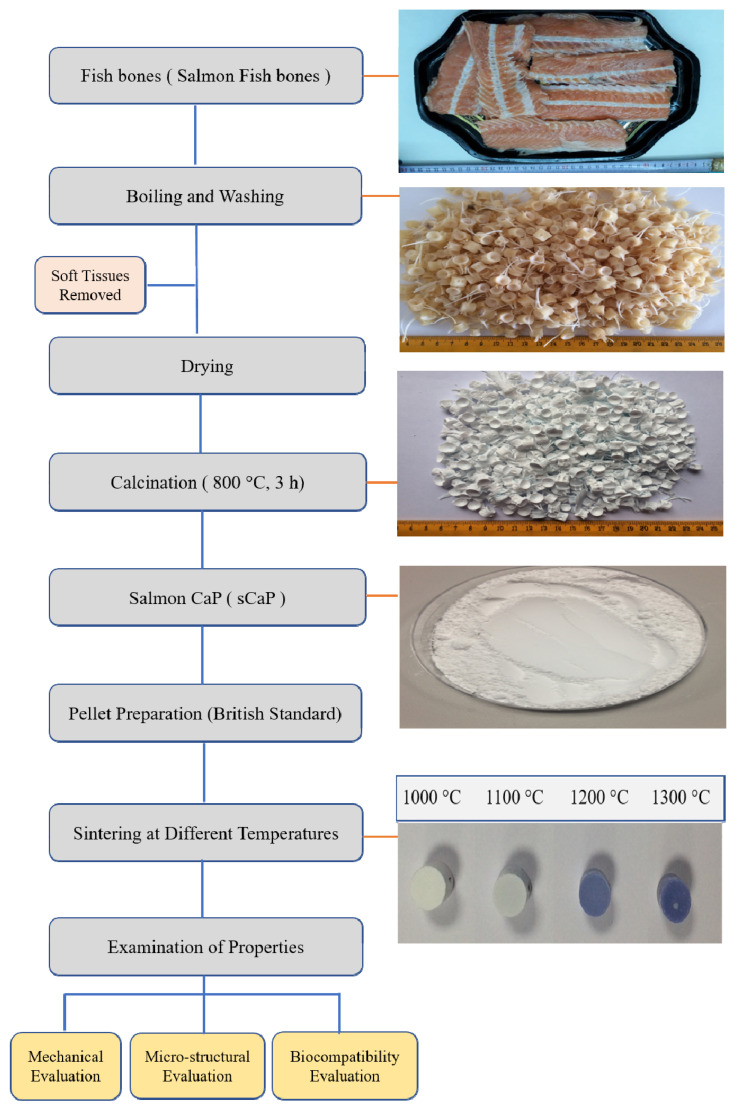
Flow diagram of natural CaP obtained from fish bones (salmon fish bones).

Salmon fish bone wastes were collected from different farms and were boiled in water at 100 °C for 1 h to remove skin and flesh marks. The cleaned bone parts were treated with 1% sodium hydroxide (NaOH) solution and washed with ultra-pure water to remove proteins, lipids and other organic impurities. Then the bones were dried [8] and calcined at 800 °C for 3 h in a custom-made cooking oven. The calcined bones were ground in RETSCH-S100 centrifugal ball mills to produce sCaP powders, which were sieved through a 63 μm sieve. The particle size distribution of the obtained sCaP powders was measured by Dynamic Light Scattering method using the HORIBA SZ-100 Z nanoparticle analyzer system. Then, fine powders with the mean particle size of about 122 nm were uniaxially pressed at 350 MPa to fabricate cylindrical pellets (11 mm in height and 11 mm in diameter) according to 7253 numbered British Standards [10]. The pressed samples were sintered at varying temperatures between 1000 °C and 1300 °C for 4 h (Nabertherm LHT 02/17 Lilienthal, Germany). Figure 1 schematically demonstrates the preparation of sCaP from salmon fish bone wastes.

### 2.2. Mechanical Properties

Vickers microhardness (HV) tests were conducted at 200 g load and 20 sec of dwell time (Shimadzu HMV-2T, Kyoto, Japan) (three samples for each sintering temperature were tested, with eight measurements made on each sample). A universal test machine DVT (Devotrans, Inc., Istanbul, Turkey) was used to perform the compression tests (2 mm/min displacement) of pelleted and sintered samples. The size and diameter data of the pellet whose compression strength were to be measured were entered into the program, and the pellet was compressed with a speed of 2 mm/min displacement. Four samples were tested for each sintering temperature, and an average value was taken. The density of the experimental samples was evaluated by the Archimedes immersion method (in water). The presenting results for density and compression tests were the average from 4 independent measurements made on different specimens. The average value of the results obtained in each experimental group was taken in Excel, and their standard deviations were calculated.

### 2.3. Characterization of the sCaP

The experimental samples were analyzed by X-ray diffraction (XRD, PANALYTICAL X’PERT PRO, the Netherlands), with CuKα radiation before and after sintering. The XRD patterns were obtained in the 2θ range of 10–90° with a step size of 0.02°. The XRD patterns were also examined by Rietveld Analysis in the High Score Plus program to proceed with quantitative analysis of the crystalline phases formed. Scanning electron microscopy (SEM) (ZEISS MA/EVO 10, Germany) was used to investigate the microstructure of the experimental samples. Elemental chemical analysis of the samples was revealed by Energy Dispersive Spectroscopy (EDX, ZEISS MA/EVO 10, Germany). Fourier transform infrared spectrometer (FTIR, JASCO FT/IR-4700, Japan) was applied in the wavenumber range 450–4000 cm^−1^ to examine the chemical structure of the samples. FTIR spectrum of commercial HA (CHA, Sigma Aldrich) was also obtained for comparison purposes. 

### 2.4. In Vitro Biocompatibility Investigation 

In vitro biocompatibility of materials was investigated by two methods: (1) MTT assay with SBF to understand whether toxic substances released from materials, (2) SEM investigations to show attachment and proliferation cells on materials. 

Saos-2 (human osteosarcoma) cell line was obtained from American Type Culture Collection (ATCC). Cells were cultured in Dulbecco’s modified Eagle medium (DMEM, Gibco) with 10% fetal bovine serum (FBS, Gibco) and 1% penicillin/streptomycin in a 5% CO_2_-humidified air incubator maintained at 37 °C. When the cells reached 80% of confluence, they were washed with PBS and trypsinized with 0.25% Trypsin-EDTA for passaging and seeding each time. The cells were used in cytotoxicity tests and SEM investigations. 

#### 2.4.1. MTT Cytotoxicity Assay

First, the conditioned medium was prepared to understand any possible toxic effect induced by possible ionic leach-out product from the samples into the medium. For this aim, a piece (~0.05 g) of sCaP was added into the 5 mL fresh medium (DMEM) and kept in a 5% CO2 incubator. After 1 day, 3 days and 7 days, the conditioned medium (or simulated body fluid (SBF)) [11] was extracted and later used in cytotoxicity tests. MTT assays were performed in 96-well plates. Saos-2 cells (about 10^5^ cells per well) were seeded onto the 4 h UV sterilized sCaP and (96-well plates) incubated for 72 h. Cell viability was measured by determining mitochondrial NADH/NADHP–dependent dehydrogenase activity, which resulted in the cellular conversion of the 3-(4, 5-dimethylthiazol-2-gl)-5-(3-carboxymethoxylphenyl)-2-(4-sulfophenyl-2H) tetrazolium salt into a soluble formazan dye. After 72 h, supernatants were removed, and 10 µL 3-{4, 5-dimethylthiazol-2yl}-2,5-diphenyl-2H-tetrazolium-bromide (MTT—5 mg/mL—Sigma) solution was added to each well. Following incubation at 37 °C for 3.5 h, they were kept dark in a humidified atmosphere with 5% CO_2_. MTT was taken up by active cells and reduced in the mitochondria to insoluble purple formazan granules according to Mosmann's study [12]. Subsequently, the supernatant was discarded, and the precipitated formazan was dissolved in dimethyl sulfoxide (100 µL per well), and optical density of the solution was evaluated using a microplate spectrophotometer (Kayto RT—2100 °C) at a wavelength of 570 nm. 

#### 2.4.2. SEM Investigations 

The sCaP samples were placed in the wells of 6-well cell culture plates and sterilized for 4 h. Saos-2 cells (about 3 × 10^5^ cells per well) were seeded in these plastic dishes and incubated for 24 h in a humidified incubator at 37 °C with 95% air and 5% CO_2_. At the end of 24 h, the media were removed, and specimens were fixed with 3% volume fraction of glutaraldehyde, subjected to graded (30–100%) alcohol dehydration and kept at −20 °C. Then they were examined with SEM [13].

## 3. Results and Discussion

**Figure 2:**
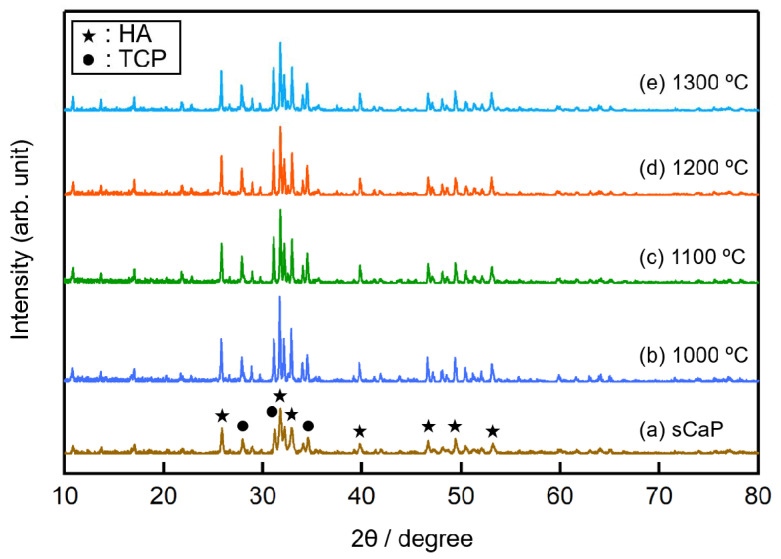
XRD patterns of sintered salmon-derived calcium phosphates (sCaP) powders (**a**) 800 °C, (**b**) 1000 °C, (**c**) 1100 °C, (**d**) 1200 °C and (**e**) 1300 °C.

The crystal phase analyses of sCaP powders sintered at different temperatures were performed by XRD, as shown in Figure 2. The XRD patterns of the calcined sCaP showed the crystalline nature of calcium phosphates mainly composed of HA and TCP as crystalline structures. Importantly, the TCP phase maintained its stability over the broad temperature range of 800–1300 °C; moreover, an amount of this phase (corresponding to JCPDS number 98-008-2984) increased with increasing temperature. HA phase amount decreased with increasing temperature. When the sintering temperature increased to 1300 °C, the powder sample became a mixture of HA and TCP phases at high temperatures [14]. The quantitative analysis of the formed crystalline phases formed is shown in Table 1. According to many papers, there are commercially available BCPs with compositions close to that obtained starting from salmon bone: 60% HA and 40% β-TCP [15].

**Figure 3:**
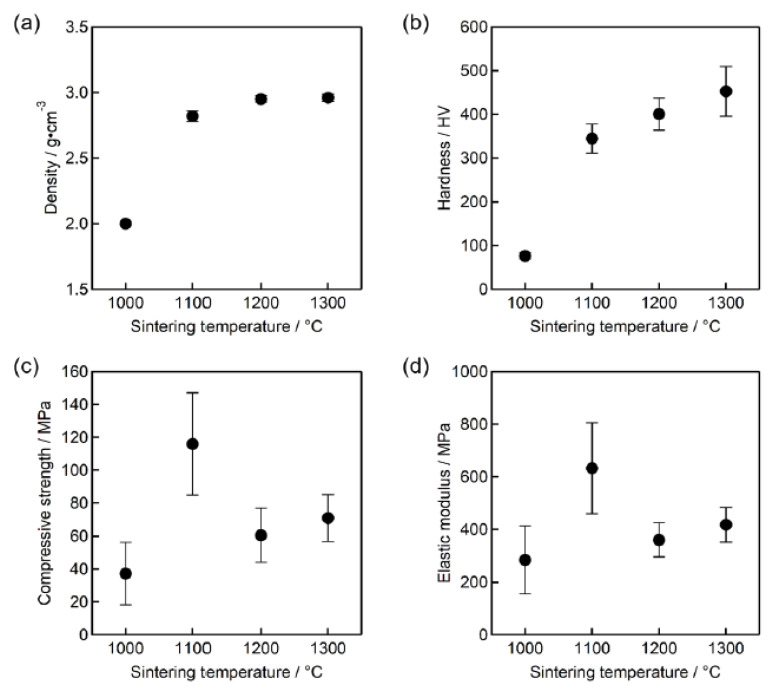
The effect of sintering temperature on the (**a**) density, (**b**) hardness, (**c**) compressive strength, (**d**) elastic modulus of sCaP.

The effect of the different sintering temperatures on density, microhardness and compressive strength are shown in Figure 3. The density of the samples (Figure 3a) increased from 2.01 ± 0.01 to 2.96 ± 0.03 g/cm^3^ when the sintering temperature was raised from 1000 to 1300 °C. Similar trends were revealed for the relationship between microhardness and sintering temperature (Figure 3b). Interestingly, there was a significant increase in the microhardness values in the temperature range of 1000–1100 °C, which might be explained by a decrease in porosity and an increase in density due to the particles’ coalescence. It was previously demonstrated that the hardness depends on structural porosity and secondary phases formed [16,17], while for HA material, it was connected with the change in density [18]. When the sintering temperature rises from 1000 to 1300 °C, there is a marked increase in Vickers microhardness from 76 ± 7 to 453 ± 57 HV. Figure 2c shows the change in compressive strength versus sintering temperature. Sintering at 1000 °C may have been insufficient (e.g., density was about 2 g/cm^3^), resulting in the lowest compressive strength compared to the samples sintered at higher temperatures. However, after sintering at 1100 °C, the compressive strength value of 116 ± 31.16 MPa was achieved, indicating that it was higher than the compressive strength of the counterparts heat-treated at 1200 and 1300 °C. It is thought that this situation may be due to phase dissociation with temperature increase. Bulut et al. added 5 and 10 wt. % commercial inert glass (CIG) to HA-Al_2_O_3_ composites to improve the microstructural and mechanical properties of HA. Composites were sintered at temperatures between 1000–1300 °C. Their mechanical properties’ results were lower than in this study’s results, despite the use of inert glass composites [19].

**Figure 4:**
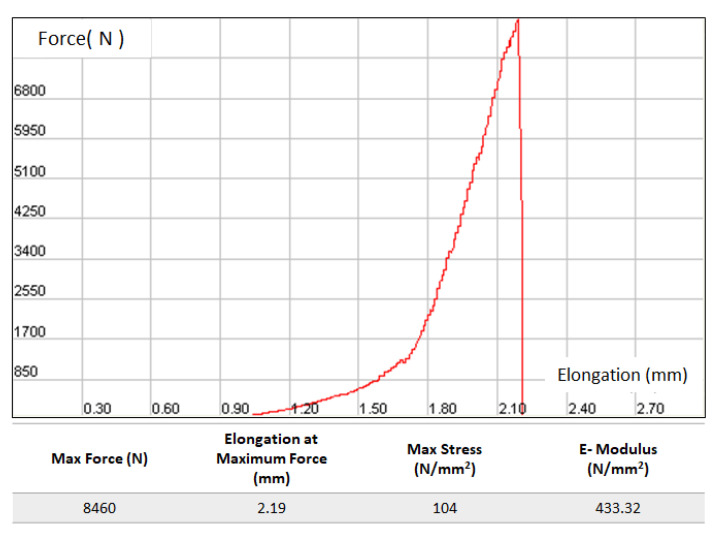
The force–elongation graph and data obtained during compression strength measurement in Devotrans Brand Compression (Press) device (sintered at 1100 °C).

Figure 3d shows the elasticity modulus of sintered samples at varying sintering temperatures, from which a very similar trend might be observed as obtained from compressive strength tests: the maximum elastic modulus of 633 MPa was recorded for sample sintered at 1100 °C, then with temperature rise to 1200 and 1300 °C, elastic modulus showed a tendency to decrease. Microstructural features and phase dissociation of the sintered samples might also contribute to that phenomenon, which will be briefly discussed in the following section. The force–elongation graph and data obtained during compressive strength measurement of a sample sintered at 1100 °C are shown in Figure 4.

**Figure 5:**
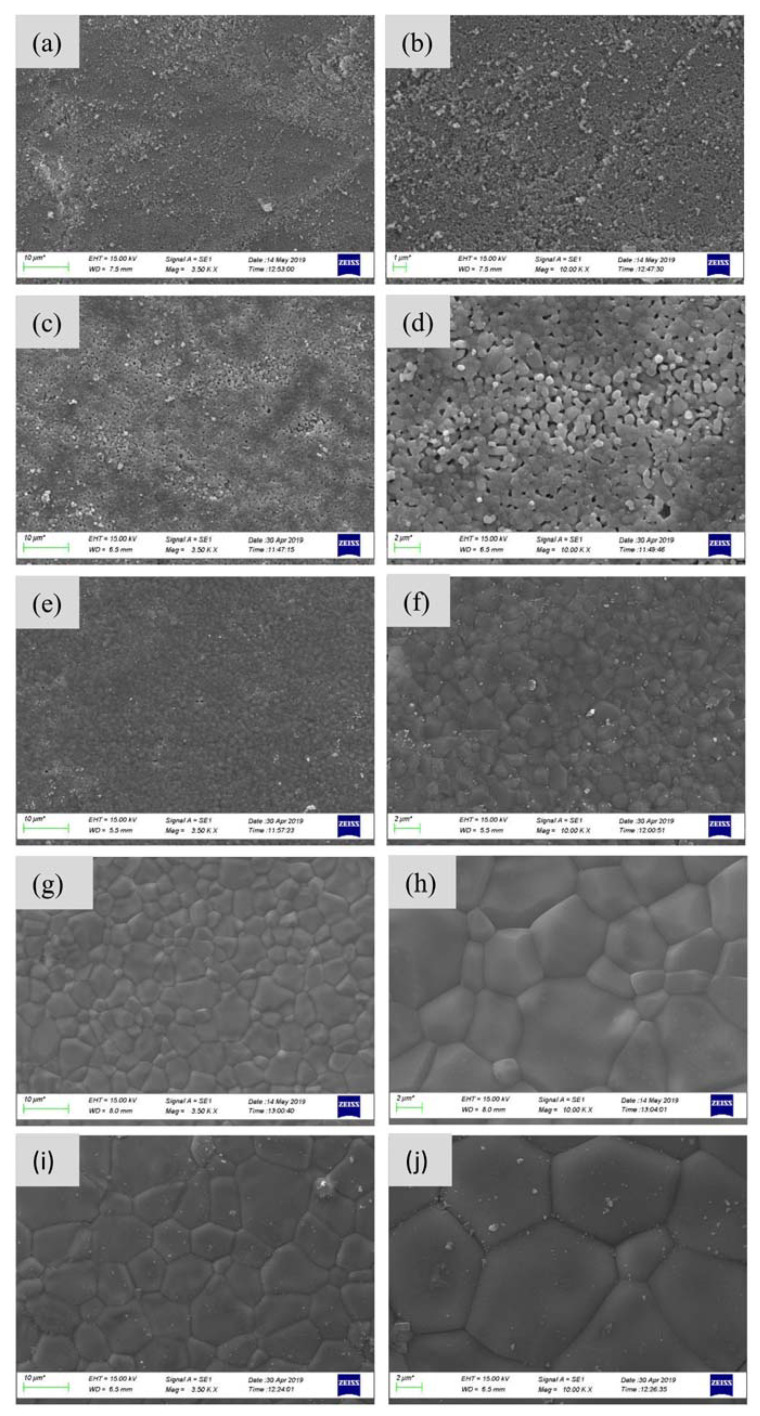
SEM micrographs of sCaP samples at different sintering temperatures; (**a**,**b**), 800 °C, (**c**,**d**) 1000 °C, (**e**,**f**) 1100 °C, (**g**,**h**) 1200 °C, and (**i**,**j**) 1300 °C.

Figure 5 shows SEM images of the sintered samples at different temperatures. Significant alterations in the morphology of the sintered samples and their particle sizes can be observed upon temperature increase from 800 to 1300 °C. In particular, with the temperature increment, the porosity decreased due to the coalescing of sCaP particles, while their grain sizes demonstrated a tendency for growth. High sintering temperatures such as 1200 and 1300 °C increase densification, and excessive heat may cause abnormal grain growth. [1]. At relatively low temperatures such as 800 and 1000 °C, there is clear evidence of an intergranular porosity between the grains (Figure 5a–d). The density of the samples increased (Figure 3a), intergranular porosity diminished, and grains were grown by coalescence for larger agglomerates (Figure 5e–j) with rising sintering temperatures. The average size of those agglomerates in the samples sintered at temperatures 1200 and 1300 °C was significantly larger than of those samples obtained at 1100 °C. The grain boundaries of fine-sized bioceramic grains have smaller imperfections, so they are stronger than large-sized grains. Generally, high crystallinity, low porosity and small particle sizes increase the mechanical properties of the material [20]. This might be an adequate explanation for the phenomenon revealed in mechanical testing, in particular in achieving the highest values of compressive strength and microhardness for the samples sintered at 1100 °C.

Energy Dispersive Spectroscopy (EDX) analysis was performed by randomly choosing 5 regions on the sample surfaces from each sintering group. As shown in Table 2, Ca, P and O were detected predominantly, but Mg level was also important. Some trace elements (Nb, Hg and Au) can be supposed to be present, but their content is at the limit of detection of EDS. Considering the tendency of bones to host a large range of oligoelements, some beneficial (Zn, Li, Sr, …) and others toxic (As, Hg, Pb, etc.), further studies will be carried out. Goto and Sasaki [21] observed that the characteristics of the HA are dependent on the nature of fish. According to literature data, heavy metals can also be found in fish bone [22], so it is important to carefully analyze the traces in the fish-derived HA. The sintered sample at 1100 °C gave a Ca/P ratio of 1.67, which was a stoichiometric ratio of HA. As the sintering temperature increased, the Ca/P ratio decreased, which can be attributed to the TCP formed by partial decomposition of HA as the sintering temperature increased, as confirmed in the XRD analysis. These ratios are obtained to see the effect of the sintering temperature on the Ca/P ratio and are approximate values because it may be possible for the peaks of different elements to overlap [23]. EDX analysis examines samples at a microscopic level, so there may be some deviations or discrepancies between the expected values [14]. It is also important to mention that EDX data are consistent with the XRD and especially with the mechanical properties. Corroborating these data, it can be concluded that the sintering effect is good enough to assure a more compact ceramic block for 1100 °C, at which temperature the ceramic model has a Ca/P ratio slightly above the stoichiometry. Unfortunately, after increasing the sintering temperature, chemical decomposition reactions occurred and the Ca/P ratio decreased to 1.52, leading to lower mechanical properties. The improvements induced by the co-existence of the two (or more) phases, a HA phase with higher stability and a TCP phase with a better resorption rate are important because they can be exploited in designing bioactivity and biodegradability and enhance bioactivity in vitro [24]. The presence of Mg^2+^ along with some traces present in the salmon fish bone is also essential in improving the desired osteogenic properties [25].

**Figure 6:**
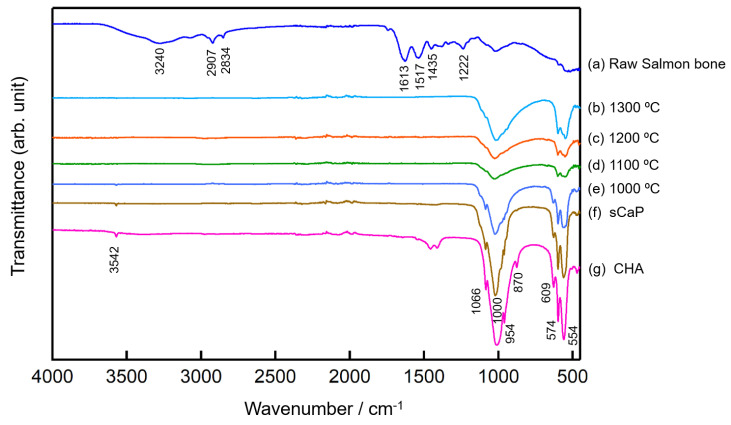
FTIR spectra; (**a**) raw salmon bone, (**b**) sintered sCaP at 1300 °C, (**c**) sintered sCaP at 1200 °C, (**d**) sintered sCaP at 1100 °C, (**e**) sintered sCaP at 1000 °C, (**f**) 800 °C sCaP and (**g**) CHA.

Figure 6 shows the FTIR spectra of raw salmon bone (fish bone) and sCaP at different sintering temperatures between 800 and 1300 °C along with commercial HA (CHA). The FTIR spectrum of raw salmon fish bones was quite different from the others, as shown in Figure 6a. Organic and mineral bands are clearly seen when comparing the FTIR graph of natural fish bone with other phosphate groups in the study. The tensile vibrations of Amide A and OH carbohydrates resulting from the protein structure in raw salmon bone are seen in the range of 3000–3400 cm^−1^. Symmetrical and asymmetrical vibrations of CH_2_, CH_3_ in the phospholipid groups are seen at 2907 cm^−1^ and 2838. Amide I at 1613 cm^−1^ and Amide II peak at 1517 cm^−1^ prove the protein structure in the awn (Figure 4) [26]. These bands in the raw fish bones show characteristic functional organic groups such as collagen, protein and oil. After salmon bones were calcined at 800 °C, peaks associated with the organic content disappeared [27]. This situation indicates that organic matter was eliminated from the bone by the calcination method. In Figure 6, typical FT-IR spectra of sCaP showed bands corresponding to OH^-^ and PO_4_^−3^. In addition, many bands such as 560, 597, 629, 960, 1011 and 1084 cm^−1^ matching the HA reference spectrum were seen. 

Absorption bands at 3542 cm^−1^ and 609 cm^−1^ are referable to structural [OH] groups (O-H), stretching and libration modes at the HA crystallite surface or crystallites. At 3570 cm^−1^, the O-H band of CHA should be observed as moisture absorption on the powder sample. The presence of [PO_4_] groups, characteristic of tetrahedral apatite structure, is proven by the characteristic of a [PO_4_] group double band at 554 cm^−1^, 574 cm^−1^ and 609 cm^−1^ [28]. The CO3^2-^ functional group disappeared at 1100 °C, and then the TCP phase began to form. These results can be also observed by characteristic shoulders in Figure 6b–f. 

**Figure 7:**
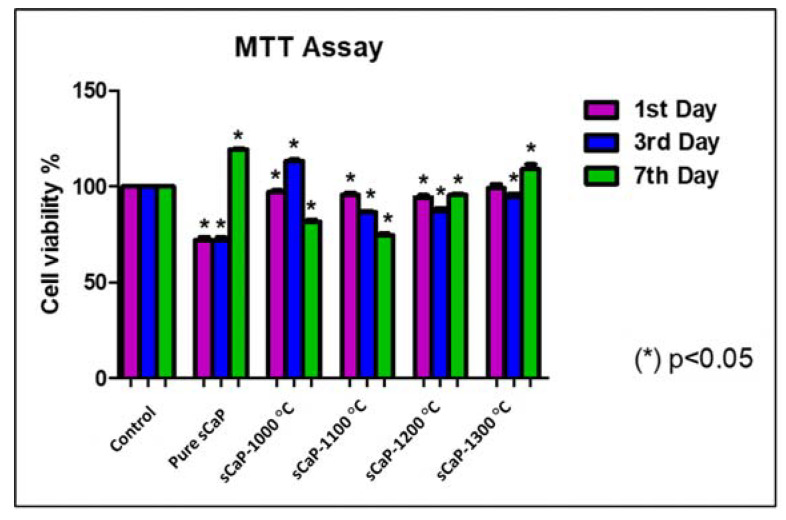
The cytotoxic effects of sCaP-1000/1100/1200/1300 °C on the viability of Saos-2 cells on different days obtained by MTT assay.

The cytotoxicity (MTT) tests shown in Figure 7 indicated that sCaP-1000/1100/1200/1300 °C had no cytotoxic effect compared to control. On the first day of the study, no changes in cell proliferation were observed. On the third day of the study, sCaP-1000 °C increased cell proliferation, and other samples were ineffective. Although the samples sintered at 1100 °C showed a similar proliferation rate with the control group on the first day, cell proliferation decreased on the 3rd and 7th days. The cells probably showed low attachment to the matrix surface. The best result was obtained in sCaP-1300 °C on the seventh day of the study. Statistically significant results are indicated by (*), and the *p*-value was calculated as *p* < 0.05 (* *p* < 0.05; data presented are mean ± SD, *n* = 3). Thus, the in vitro biocompatibility tests demonstrated that these composites had suitable cytocompatibility, and they can be recommended for further developments for biomedical applications.

**Figure 8:**
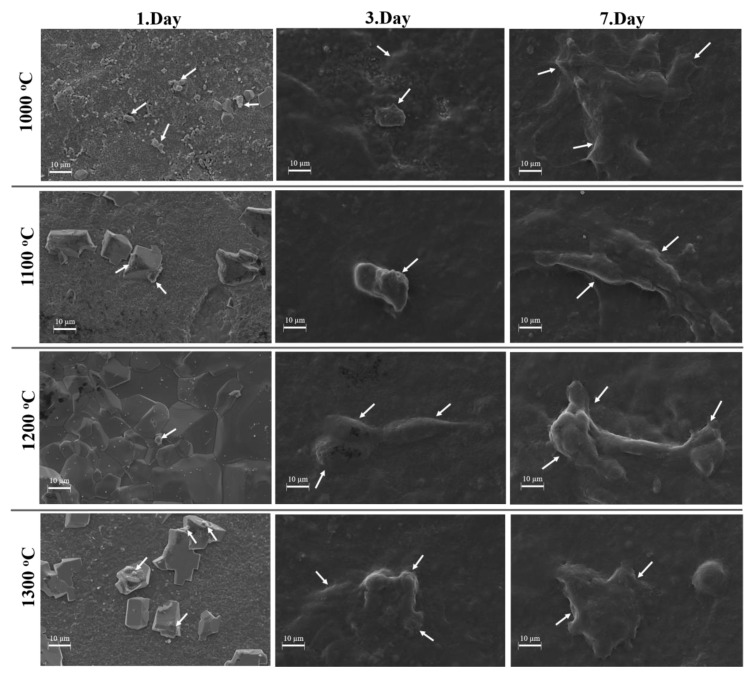
Time-dependent cell attachment and proliferation of sCaP at 1., 3. and 7. days at 1000 °C, 1100 °C, 1200 °C and 1300 °C sintering temperatures in SEM images.

The Saos-2 (human osteosarcoma) cells were cultured on the sCaP composites, and they were fixed at 1, 3, and 7 days then cell attachment and proliferation were examined (Figure 8). In the SEM image, it was observed that the cell clusters on sCaP composites obtained in three different temperatures between 1000 and 1300 °C were attaching and proliferating. Thus, it has been verified that sCaP has nontoxic and significant proliferation properties, as expected based on literature data and traditional use.

## 4. Conclusions

Fish bones are usually considered useless; however, these wastes are a rich source of calcium phosphate. Reuse of fish bones in this way can reduce waste costs to the environment and the risk of environmental pollution. nHA was obtained from salmon fish bones wastes by using the calcination method. Typical stoichiometric HA groups and TCP were observed in the FTIR and XRD analysis. With increasing sintering temperature, the amount of TCP increased, while the amount of HA decreased. The average grain size and density increased with sintering temperature. EDX analysis results show that while Ca/P ratio approached stoichiometric HA in sintered samples at 1100 °C, as the sintering temperature increased, Ca/P ratio decreased due to the increasing amount of TCP. In vitro biocompatibility test and SEM results revealed that sCaP composites could be available clinically for bone repair and regeneration applications. These findings indicate that natural CaP produced from salmon bones constitutes a cost-effective and environmentally friendly CaP source, maintaining the normal oligoelements of the natural bones, such as Mg^2+^, and according to the literature, this is important because it will assure better osteointegration and biocompatibility. It is important to mention that further works will be necessary to analyze the presence of the trace elements (heavy metals as well as the level of the desired oligo-elements) in the fish-derived CaP.

## Figures and Tables

**Table 1:** Quantitative analysis of the crystalline phases formed according to JCPDS code.

	98-008-2984	98-005-2691	98-007-7966	98-006-0425	98-004-9808	98-004-0602	98-005-2686
	TCP (Ortho-Phosphate) (%)	HA (%)	HA (%)	HA (%)	HA (%)	HA (%)	HA (%)
sCaP	34.6	44.6	20.8				
1000 °C	38.1	37.2	24.7				
1100 °C	41			59			
1200 °C	45.6				53.2	1.1	
1300 °C	46.5		0.4				53.1

**Table 2:** EDX analysis and Ca/P ratios of sintered sCaP.

Sintering Temperature (°C)	Element Composition (Atomic%)				
	Ca	O	P	Mg	Ca/P Ratio
800	28.68	52.41	17.58	1.50	1.63
1000	37.12	51.14	21.40	1.58	1.73
1100	35.86	60.28	21.41	1.60	1.67
1200	27.71	50.73	18.19	1.72	1.52
1300	30.05	48.18	19.65	1.93	1.52
